# A genetic screen for increasing metabolic flux in the isoprenoid pathway of *Saccharomyces cerevisiae*: Isolation of *SPT15* mutants using the screen

**DOI:** 10.1016/j.meteno.2016.05.004

**Published:** 2016-05-27

**Authors:** M. Wadhwa, A.K. Bachhawat

**Affiliations:** Department of Biological Sciences, Indian Institute of Science Education and Research, Mohali, S.A.S Nagar, Punjab 140306, India

**Keywords:** T, TEF promoter, C, CYC promoter, GGPPS, Geranylgeranyl diphosphate synthase, PSY1, phytoene synthase, CRTI, phytoene dehydrogenase, Metabolic engineering, Carotenoids, Isoprenoids, α-Farnesene, *Rhodosporidium toruloides*, *SPT15*

## Abstract

A genetic screen to identify mutants that can increase flux in the isoprenoid pathway of yeast has been lacking. We describe a carotenoid-based visual screen built with the core carotenogenic enzymes from the red yeast *Rhodosporidium toruloides.* Enzymes from this yeast displayed the required, higher capacity in the carotenoid pathway. The development also included the identification of the metabolic bottlenecks, primarily phytoene dehydrogenase, that was subjected to a directed evolution strategy to yield more active mutants. To further limit phytoene pools, a less efficient version of GGPP synthase was employed. The screen was validated with a known flux increasing gene, *tHMG1*. New mutants in the TATA binding protein *SPT15* were isolated using this screen that increased the yield of carotenoids, and an alternate isoprenoid, α-Farnesene confirming increase in overall flux. The findings indicate the presence of previously unknown links to the isoprenoid pathway that can be uncovered using this screen.

## Highlights

• *R. toruloides* carotenogenic enzymes yield high β-carotene levels in *S. cerevisiae.*
• Phytoene dehydrogenase mutant (*Rt*CRTI_A393T_) isolated through directed evolution.
• Preventing phytoene buildup by modulating the precursor enzyme GGPP synthase.
• Genetic visual screen for increased flux in isoprenoid pathway validated by *tHMG1.*
• Isolation of novel *SPT15* mutants that show increased β-carotene and α-Farnesene.

## Graphical abstract

**Figure:**
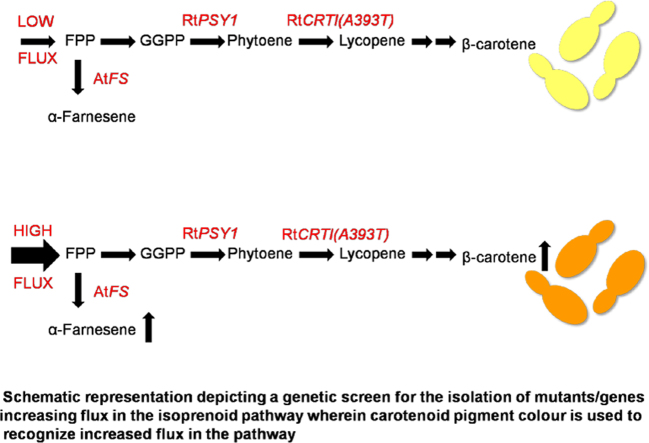

## 1 Introduction

Isoprenoids or terpenoids represent the largest class of natural products with more than 40,000 known structures (Bohlmann and Keeling, 2008). Many of these terpenoids are of immense commercial value. Their biosynthesis in heterologous hosts offers an alternative to the chemical synthesis or the extraction from their natural sources (Farhi et al., 2011, Herrero et al., 2008, Keasling, 2010). *Saccharomyces cerevisiae* is one of the choice organisms as heterologous host for terpenoids (Hong and Nielsen, 2012, Nevoigt, 2008).To increase the yield of isoprenoids in yeast, previous efforts have focused on manipulating the mevalonate pathway. Using known information about the mevalonate-isoprenoid pathway, three potential targets were identified as being potential bottlenecks for isoprenoid biosynthesis, HMG-CoA reductase (*HMG1*), the transcription factor *UPC2* and the ergosterol branch point *ERG9*. Using truncated *tHMG1* (that lacks feedback regulation) (Asadollahi et al., 2010, Gardner and Hampton, 1999, Ro et al., 2006, Westfall et al., 2012, Zhou et al., 2012), a hyperactive transcription factor *upc2-1* (that increases expression of the mevalonate pathway) (Ro et al., 2006, Westfall et al., 2012), or reduced expression of *ERG9* (that prevents isoprenoids from branching off) (Asadollahi et al., 2010, Babiskin and Smolke, 2011, Paradise et al., 2008, Ro et al., 2006, Westfall et al., 2012), increased flux has been demonstrated and the yield of isoprenoids further increases when these different mutations are combined. However, in the cell, metabolic pathways are interconnected and tightly regulated (Szappanos et al., 2011), and it is possible that besides the mevalonate pathway genes, there may be other genes which affect directly or indirectly the yield of carotenoids or other terpenoids produced in yeasts. To identify these, a good genetic screening method is required. As carotenoids are coloured compounds, their production by yeast cells provides a good visual phenotype, and this has been extensively exploited in the past (Mitchell et al., 2015, Schmidt-Dannert et al., 2000, Wang et al., 2009, Xie et al., 2014). However, surprisingly, despite their extensive use in a variety of different screens and assays, their development as a measure of isoprenoid flux has remained unsuccessful so far.

A few groups have attempted to increase the metabolic flux in the isoprenoid pathway using this carotenoid based visual screen using the carotenogenic enzymes from *Xanthophyllomyces dendrorhous* (Ozaydin et al., 2013, Verwaal et al., 2007, Yuan and Ching, 2014). However these studies have met with limited success. It was observed that upon increasing the flux in this pathway through known flux increasers such as *tHMG1*, a decrease (rather than an increase) in pigmentation was observed (Verwaal et al., 2007, Yuan and Ching, 2014). Estimation of carotenoids revealed that the decrease was most likely due to accumulation of the colourless intermediate, phytoene which masked any increase in colour due to higher β-carotene (Verwaal et al. 2007). A visual carotenoid based screen has also been employed to screen the yeast deletion collection to identify gene deletions that could improve isoprenoid production (Ozaydin et al. 2013). Although the study succeeded in obtaining deletion mutants with more β-carotene, it did not appear to be a validated screen for isoprenoids since the higher pigmentation yielding deletion mutants did not yield increased levels of an alternate isoprenoid, bisabolene.

The red yeasts belonging to the *Rhodotorula* spp., *Rhodosporidium* spp. and *Sporobolomyces* spp. have an intense red colour and are considered to be the yeasts with the highest β-carotene levels (Mata-Gomez et al., 2014). In addition to β-carotene, these yeasts produce the carotenoids- torulene and torularhodin. Owing to the high production of carotenoids from these yeasts, the possibility that the enzymes from these organisms might have evolved to be more efficient seems a likely possibility. In attempting to develop a genetic screen for isoprenoid/carotenoid production in *Saccharomyces cerevisiae*, we have sought to use enzymes from these yeasts in place of *X. dendrorhous.* Based on the recently released genome sequences of *Rhodosporidium toruloides* by multiple groups (Kumar et al., 2012, Zhu et al., 2012) we identified and carried out codon-optimised expression of the genes for the core biosynthetic carotenogenic enzymes upto β-carotene from *R. toruloides* into *S. cerevisiae*. Although the core carotenogenic enzymes of *R. toruloides* were more efficient, they still lacked sufficient capacity of pulling increased flux in the pathway through it, and a metabolic bottleneck at phytoene dehydrogenase, *Rt*CRTI, was identified as the rate limitng step. *Rt*CRTI was subjected to a directed evolution strategy and from a mutant library variant enzymes with enhanced activity were isolated. As phytoene levels needed to be further decreased, we used a less efficient version of the precursor enzyme GGPP synthase on a weaker promoter to relieve phytoene buildup to eventually yield a combination that could function as a genetic screen, as validated by over expression of *tHMG1* in this background. The developed screen enabled the identification of mutants of TATA binding protein *SPT15*, that increased yields of β-carotene. The isolated *spt15* mutants could also enhance the levels of an alternate isoprenoid, the sesquiterpene α-Farnesene suggesting that the mutants were in fact enhancing isoprenoid flux and were not exclusive to carotenoids. These results, which describe and validate a carotenoid-based screen for isoprenoid flux in yeast, are described in this report.

## 2 Materials and methods

### 2.1 Plasmid vectors, cloning of genes and transformation

The yeast centromeric plasmids p416TEF, pRS313TEF, pRS314TEF, pRS315TEF as well as the same series with the CYC promoter were used for cloning and expression of carotenogenic genes. pRS313TEF, pRS314TEF and pRS315TEF were constructed by excising the MCS and TEF promoter regions from p416TEF plasmid and cloning into pRS313, pRS314 and pRS315 respectively. The genes for Geranylgeranyl diphosphate (GGPP) synthase (*Rt*GGPPS), Phytoene synthase (*Rt*PSY1) and Phytoene dehydrogenase (*Rt*CRTI) of *R. toruloides* were codon optimised by using EnCor Biotechnology Inc. (http://www.encorbio.com/protocols/Codon.htm) software and custom synthesised by GenScript USA. These genes are cloned in pRS315TEF, p416TEF and pRS314TEF respectively. *Rt*GGPPS was cloned at the *Xba*I and *Bam*HI sites of pRS315TEF to construct pRS315TEF- *Rt*GGPPS, *Rt*PSY1 was cloned at *Bam*HI and *Xho*I sites of p416TEF to yield p416TEF- *Rt*PSY1 while *Rt*CRTI is cloned at *Bam*HI and *Sal*I site to construct pRS314TEF-*Rt*CRTI. For over expression of truncated HMG CoA reductase 1 (*tHMG1*), the C-terminal catalytic region (1575 bp) was amplified from *S. cerevisiae* genomic DNA using *tHMG1*-FP and *tHMG1*-RP and the amplified PCR product was cloned at the *Bam*HI and *Xma*I sites of pRS313TEF to construct pRS313TEF-*tHMG1*. For construction of p416CYC- *Rt*PSY1, p416TEF-*Rt*PSY1 is digested with *Sac*I and *Bam*HI to excise the TEF promoter and ligated with *Sac*I and *Bam*HI digested CYC1 promoter from p414CYC1 vector. pRS314CYC-*Rt*CRTI was constructed similarly. pRS315CYC- *Rt*GGPPS was constructed from pRS315TEF-*Rt*GGPPS by digesting with *Xba*I and *Sac*I to excise the TEF promoter and ligated with *Xba*I and *Sac*I digested CYC1 promoter of p414CYC1 vector. *SPT15* was amplified from *S. cerevisiae* ABC 276 strain by using the forward and reverse primers and cloned in the *Bam*HI and *Xho*I sites of pRS313TEF. The cDNA for α- Farnesene synthase (Locus AT4G16740 and clone no. U88221) from *Arabidopsis thaliana* was obtained from TAIR database, USA. It was PCR amplified and subcloned in *Xba*I and *Bam*HI site of pRS315TEF to make the construct pRS315TEF-*At*FS. All these constructs were transformed into *S. cerevisiae* strain (ABC276) by Lithium acetate method (Sambrook, 1989). All the primers and plasmids constructed in this study are indicated in Table S1 and S2.

### 2.2 Strains and media

*Escherichia coli* strain DH5α was used as cloning host. *S. cerevisiae* strains CEN. PK2-1C (Euroscarf accession no. 30000 A) -*MAT a, ura 3-52,trp 1-289, leu2-3_112, his3*Δ*1, MAL 2-8*^*c*^, SUC2 and ABC 276 which is a derivative of S288c strain with genotype *MAT α ura 3-52 leu2*Δ*1 his3*Δ*200 trp1 lys2-801* are used in this study. The strain was derived from tetrad analysis of diploids made between BJ5418 and BJ5458 strains which are obtained from the Beth Joan laboratory. These strains were maintained on yeast extract, peptone and dextrose (YPD) media. For culturing yeast-synthetic defined media (SD) containing yeast nitrogen base (YNB) without ammonium sulphate 0.15% (w/v) and amino acids supplemented with appropriate amino acids and 0.5% (w/v) ammonium sulphate and 2% (w/v) d-glucose was used.

### 2.3 Extraction of carotenoids and analysis by HPLC

Extraction of carotenoids were carried out as described earlier (Moline et al., 2012) with some modifications. Essentially, yeast cells were grown in 100 mL SD media supplemented with appropriate amino acids and grown at 30 °C with shaking (250 rpm). After five days, cells were harvested and washed with deionized water and kept at −20 °C. To the frozen pellet was added 3 mL of Dimethyl sulphoxide (DMSO), vortexed for 1 min and incubated at 55 °C in the water bath for 1 h. 1 g 0.50–0.75 mm glass beads were added, and cells were broken using glass bead beater. Cells were centrifuged to remove the cell debris. Acetone was added to the pellet, vortexed and centrifuged and the process repeated till the pellet becomes colourless. The acetone and DMSO fractions were mixed with an equal amount of Hexane. The coloured hexane layer was collected after separation of two layers. The hexane layer was washed with distilled water and then with brine solution twice. The coloured hexane layer was collected. The solvent was evaporated under rotary evaporator to dryness in dim light and was dissolved in 1 mL hexane for analysis by high performance liquid chromatography (HPLC). HPLC separation and quantification was performed on Waters System using C_18_− 5 μm intersil ODS-P, 250×4.6 mm column (LCGC) using solvent acetonitrile:methanol:2-propanol (85:10:5 v/v) with flow rate 1 mL/min at 32 °C. Separated carotenoids were detected by photodiode array detector. Quantification of carotenoids was done using a standard curve prepared for β-carotene, lycopene and phytoene. Standards for β-carotene and lycopene were obtained from Sigma Aldrich, India and phytoene were obtained from CaroteNature GmbH, Switzerland. Standards of β-carotene, lycopene and phytoene were dissolved in hexane. The concentration of standard solution of β-carotene, lycopene were calculated using extinction coefficient (A^1%^_1cm_) of 2590 (g/100 mL)^−1^ cm^−1^ at 450 nm and (A^1%^_1cm_) of 3450 (g/100 mL)^−1^ cm^−1^ at 470 nm in hexane respectively and the concentration of phytoene was calculated using extinction coefficient (A^1%^_1cm_) of 750 (g/100 mL)^−1^ cm^−1^ at 285 nm in hexane/2% CH_2_Cl_2_. The concentration of β-carotene, lycopene and phytoene in samples were expressed in microgram per gram dry cell weight (microgram/gram DCW). Data represented in form of standard mean error of at least two independent experiments. For estimating the dry cell weight, samples were kept at 80 °C in an oven for 48 h and their dry weight were determined.

### 2.4 Identification and quantification of α-Farnesene

*S. cerevisiae* ABC 276 was transformed with pRS315TEF-*At*FS. Transformants were grown in SD media containing appropriate amino acids. Secondary culture was inoculated at 0.05 OD_600_ and when OD_600_ reaches to 0.6–0.8, culture was overlaid with 10% dodecane. After 48 h, the dodecane phase of the two- phase culture was collected by centrifugation of culture at 6000 rpm for 5 min. 1 μL of dodecane phase was subjected to GC-FID analysis. Samples were injected at a split ratio of 1:10. The oven temperature was initially held at 80 °C for 1 min and was increased at a rate of 10 °C/min to 250 °C where it was held for 1 min. Carrier gas was nitrogen. And the temperature of detector was maintained at 260 °C. All the conditions used for GC analysis was followed from (Wang et al., 2011). Standard curve of trans β-Farnesene was prepared using GC- FID. Trans β-Farnesene (Cat. 73492) from Sigma Aldrich, India was used as standard.

### 2.5 *In vitro* mutagenesis

Random mutagenesis *in vitro* was performed on the purified plasmids by hydroxylamine as described earlier (Rose and Fink, 1987). The average number of mutations obtained from hydroxylamine mutagenesis was approximately 1 per kb.

### 2.6 Dilution spotting for growth and colour visualisation

Yeast cells were grown overnight in SD media supplemented with appropriate amino acids, reinoculated in fresh media at 0.1 OD_600_ and grown to 0.6–0.8 OD_600_ Yeast cells were harvested, washed with deionized water and resuspended at different dilutions. 10 μl of different dilutions at 0.2, 0.02, 0.002 and 0.0002 OD_600_ are spotted on SD plate supplemented with appropriate amino acids. Only confluent spot of 0.2 OD_600_ dilution are shown.

### 2.7 Modelling of phytoene dehydrogenase

Modelling of phytoene dehydrogenase of *R. toruloides* was performed using Phyre2 (http://www.sbg.bio.ic.ac.uk/phyre2). Phytoene dehydrogenase from *Pantoea ananatis* (PDB Id-4DGK) was used as a template for modelling.

### 2.8 Sequence accession numbers

The codon optimised and custom synthesised genes – GGPP synthase (*Rt*GGPPS), Phytoene synthase (*Rt*PSY1) and phytoene dehydrogenase (*Rt*CRTI) were submitted to Genbank database and have the following accession numbers KU041640, KU041641 and KU041642 respectively.

## 3 Results

### 3.1 Identification and reconstruction of the core carotenoid biosynthetic pathway genes of *Rhodosporidium toruloides* into *Saccharomyces cerevisiae*

**Fig. 1:**
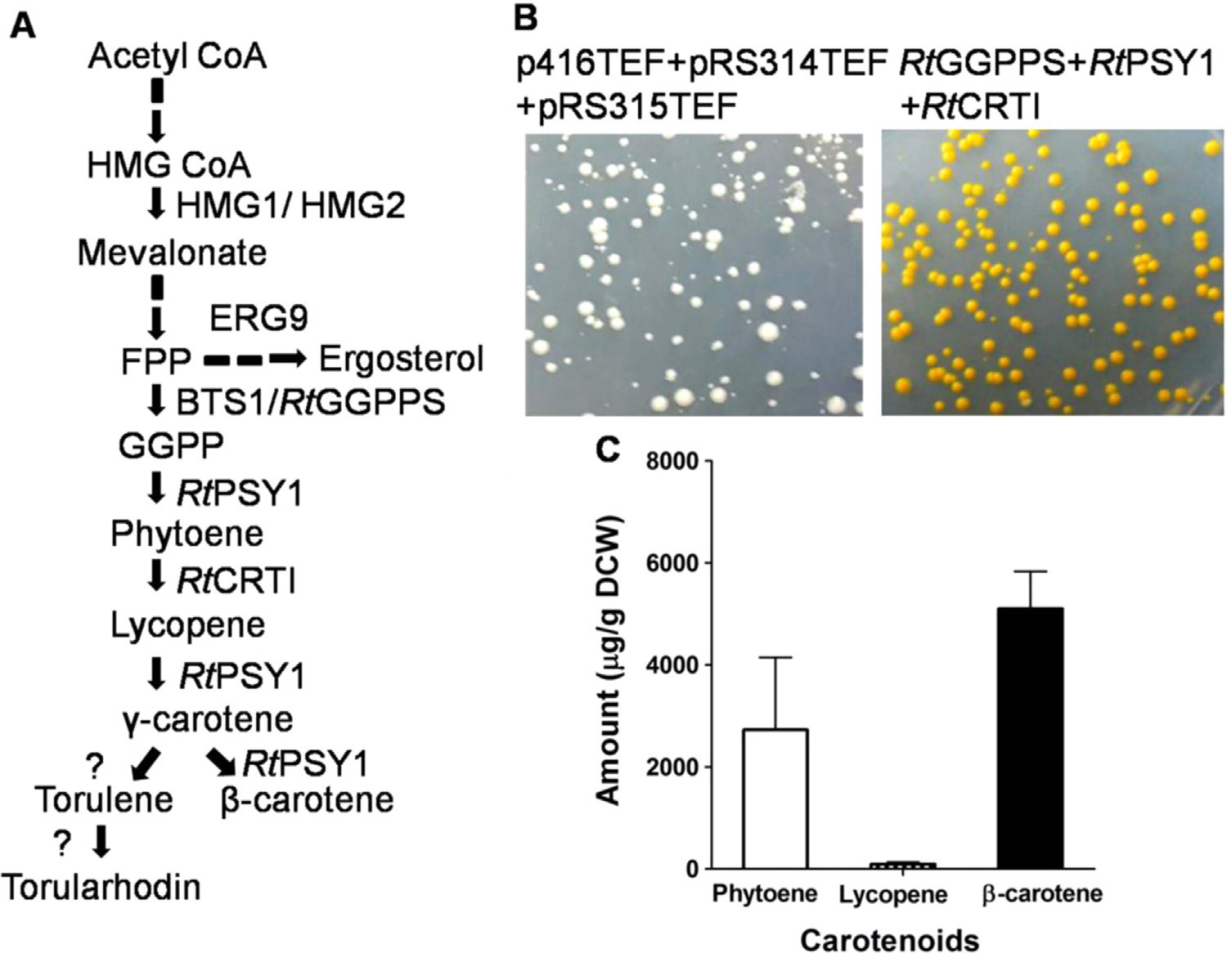
*Rhodosporidium toruloides* carotenogenic genes in *S. cerevisiae* (A) Schematic representation of proposed carotenogenic pathway in *R. toruloides* (B) Functional expression of core carotenogenic genes in *S. cerevisiae* (C) Amount of key carotenoids.

The red yeasts that include *R. toruloides* are amongst the highest producers of β-carotene (Mata-Gomez et al., 2014). We have therefore sought to reconstruct the core carotenoid pathway of *R. toruloides* in *S. cerevisiae.*The core pathway involves three enzymes- GGPP synthase, Phytoene synthase and Phytoene dehydrogenase. Expression of these genes have been predicted to produce Lycopene, γ-carotene and β-carotene (Fig. 1A).

Using the genome sequence of this yeast that we recently described along with others (Kumar et al., 2012, Zhu et al., 2012) we identified the putative genes that code for Geranylgeranyl diphosphate synthase (*Rt*GGPPS), Phytoene synthase (*Rt*PSY1) and Phytoene dehydrogenase (*Rt*CRTI). The putative gene and protein sequences were retrieved and genes with ORF numbers as *Rt*GGPPS (RHTO_02504), Phytoene synthase (RHTO_04605) and Phytoene dehydrogenase (RHTO_04602) were obtained.

The GGPP synthase gene encodes a protein of 359 aa with 62% similarity (E -value 2e^−112^) to GGPPS from *Xanthophyllomyces dendrorhous.* The Phytoene synthase gene encodes a protein of 612 aa with 45% similarity (E -value 4e^−76^) to the phytoene synthase of *X. dendrorhous.* The predicted Phytoene dehydrogenase of *R. toruloides* is 610 aa in length. However, the protein appeared to have an extra N-terminal of 56 amino acids as compared to the phytoene dehydrogenase of *X. dendrorhous* and other phytoene dehydrogenases (Data not shown). It thus appeared that this extra N-terminal may be a consequence of a mis-annotation of the start site, and we therefore only considered the genic region that corresponded to the remaining 554 aa. The protein has 68% similarity (E-value 2e^−176^) to the Phytoene dehydrogenase from *X. dendrorhous.*

All three genes contained multiple introns, and as *R. toruloides* has a high G-C content compared to *S. cerevisiae*, we opted to custom synthesise the cDNAs for these enzymes after codon optimisation for expression in *S. cerevisiae.*

The *Rt*GGPPS, *Rt*PSY1 and *Rt*CRTI cDNAs were cloned in yeast single copy centromeric expression vectors under the TEF promoter and the CYC terminator. We chose to use the centromeric vectors owing to their greater stability. The genes were cloned in pRS315TEF, p416TEF and pRS314TEF, respectively. These constructs were transformed in *S. cerevisiae* ABC 276 strain and transformants were selected on SD-ura-leu-trp plates. Expression of these genes produced a deep orange colour in yeast (Fig. 1B). Estimation of carotenoids using HPLC showed that expression of *Rt*GGPPS, *Rt*PSY1 and *Rt*CRTI were able to produce β-carotene (5105±732 µg/g DCW), phytoene (2727±1421 µg/g DCW) and negligible amounts of lycopene (95±37 µg/g DCW) based on comparison with retention time of available authentic carotenoid standards (Fig. 1C). We have also obtained four unknown peaks in the HPLC chromatogram whose identities are yet to be determined (Fig. S1). These unknown compounds have relatively small peak area as compared to β-carotene at 450 nm wavelength. Based on the predicted pathway, they may include either torulene or γ-carotene, but this was not determined.

In previous studies in which the carotenogenic genes of *X. dendrorhous* (in episomal plasmids) were expressed in *S. cerevisiae*, high levels of phytoene as compared to levels of lycopene and β-carotene were detected (Verwaal et al., 2007). In contrast, using the *R. toruloides* genes we observed β-carotene as the major fraction of total carotenoids with lower amount of phytoene and negligible amount of lycopene, although a similar deep orange coloured colony as observed by Verwaal et al. (2007),was observed in our case also. This preliminary analysis suggested that opting for genes from *R. toruloides* appeared advantageous since it led to low levels of intermediates (i.e. phytoene and lycopene), and 20 fold higher yields of β-carotene as compared to reported previously (Verwaal et al., 2007).

### 3.2 Identification of the rate-limiting step in the carotenoid production through a combinatorial approach of weak and strong promoters driving expression of the *Rt*GGPPS, *Rt*PSY1 and *Rt*CRTI genes

**Fig. 2:**
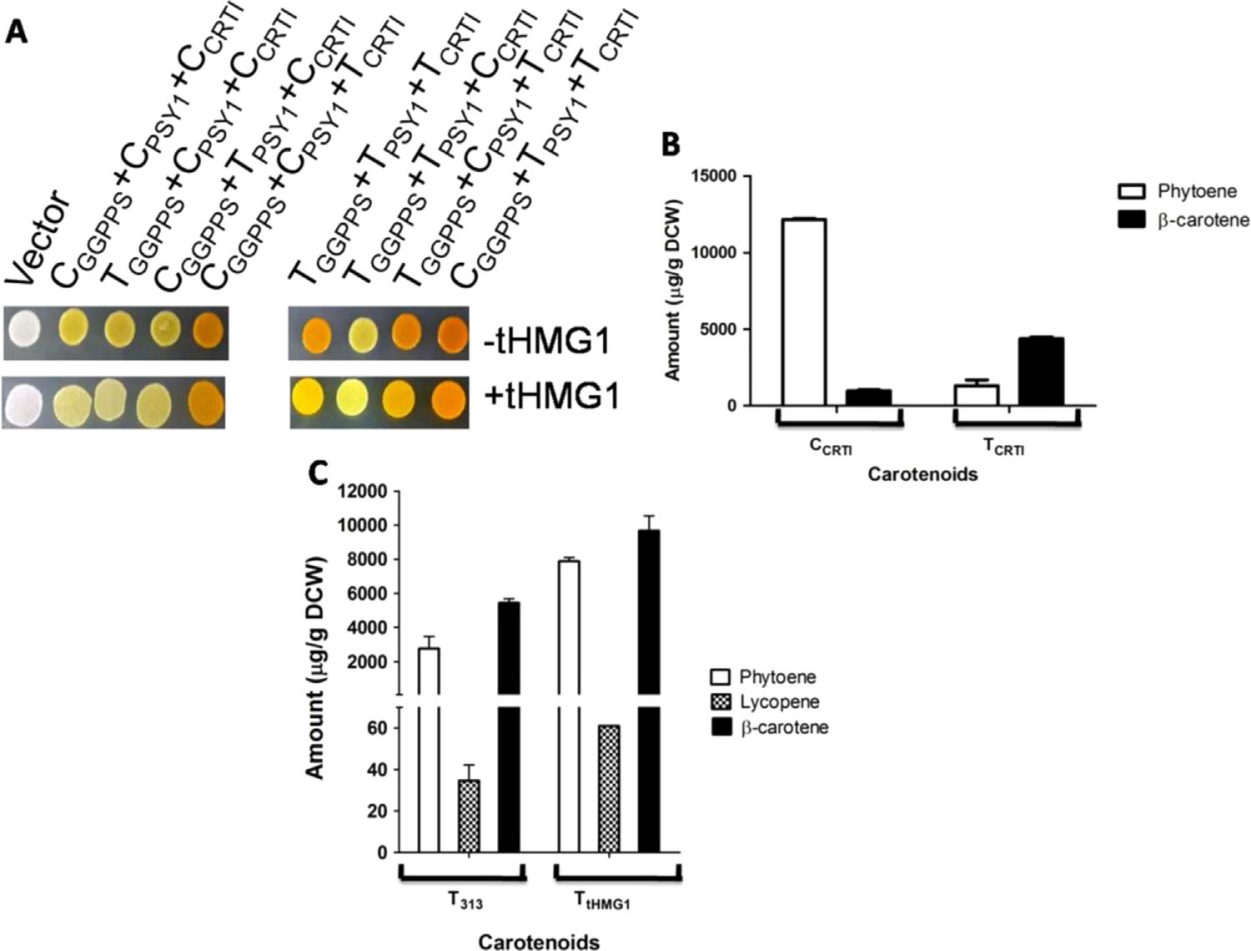
Evaluation of isoprenoid flux. (A) On the pigmentation level of different promoter combination strains with and without *tHMG1* (spot containing 4* 10^4^ cells) (B) Amount of carotenoids when *Rt*CRTI is under weak (CYC) and strong (TEF) promoter. (C) Amount of key cartenoids when *R. toruloides* carotengenic (Rt) genes are expressed with and without *tHMG1* (under TEF promoter).

To use carotenoid levels as a visual measure of increased flux in the isoprenoid pathway, we needed to identify if there were any rate-limiting steps that were leading to metabolic bottlenecks in the pathway. In plants, phytoene synthase is known to be rate-limiting (Qin et al., 2011). In contrast, when *Xanthophylomyces* enzymes were over-expressed in *S. cerevisiae*, phytoene dehydrogenase was found to be rate-liming (Verwaal et al., 2007). We were keen to identify if there were any rate-limiting steps when enzymes from the high carotenoid producing *R. toruloides* were being used and were also codon-optimised for expression. Towards this objective we cloned and expressed *Rt*GGPPS*, Rt*PSY1*, Rt*CRTI under the weak CYC promoter and the strong TEF promoter (both with the CYC terminator). A *S. cerevisiae* strain was separately transformed with different TEF and CYC promoter combinations of *Rt*GGPPS*, Rt*PSY1and *Rt*CRTI gene constructs. Pigmentation intensity of the colony was used as the readout. We observed that the plasmid combinations that produced more colour were those that contained phytoene dehydrogenase (*Rt*CRTI) expressed under a strong constitutive promoter (Fig. 2A). These plasmid combinations were: TEF_GGPPS_+TEF_PSY1_+TEF_CRTI_, CYC_GGPPS_ +CYC_PSY1_+TEF_CRTI_, CYC_GGPPS_+TEF_PSY1_+TEF_CRTI_ and TEF_GGPPS_+CYC_PSY1_+TEF_CRTI_. Conversely, the combinations that produced the least colour were when *Rt*CRTI was expressed under the weak promoter. These combination strains are as CYC_GGPPS_+ CYC_PSY1_+CYC_CRTI_, TEF_GGPPS_+TEF_PSY1_+CYC_CRTI_, TEF_GGPPS_+CYC_PSY1_+CYC_CRTI_ and CYC_GGPPS_+TEF_PSY1_+ CYC_CRTI_ (Fig. 2A). These experiments suggested that phytoene dehydrogenase (encoded by *Rt*CRTI) was a rate-limiting enzyme and was confirmed by chemical analysis (Fig. 2B). This rate-limiting step might interfere with the development of the use of carotenoid as a visual screen for metabolic flux in this pathway. With phytoene dehydrogenase limiting, increasing the flux would lead to accumulation of colourless intermediate, phytoene which by non-linear flux/pigmentation relationships hinders pigmentation in visual screens.

To examine this issue we over-expressed the truncated catalytic domain of *HMG1* (*tHMG1*) which is known to increase the flux in the pathway, in these different promoter combination strains. The over expression of *tHMG1* in these different strains did not show an increase in colour despite an expected increase in flux in the isoprenoid pathway and further suggested a masking effect due to accumulated phytoene (Fig. 2A). Chemical analysis of the carotenoids accumulating in the TEF_GGPPS_+TEF_PSY1_+TEF_CRTI_ strain revealed that with over expression of *tHMG1*, there is 2.8 fold increase in levels of phytoene, but only 1.8 fold increase in the levels of β-carotene confirming the metabolite accumulation at the phytoene dehydrogenase step (Fig. 2C).

### 3.3 Directed evolution of the *R. toruloides* phytoene dehydrogenase using a pigmentation screen for the isolation of catalytically efficient mutants

**Fig. 3:**
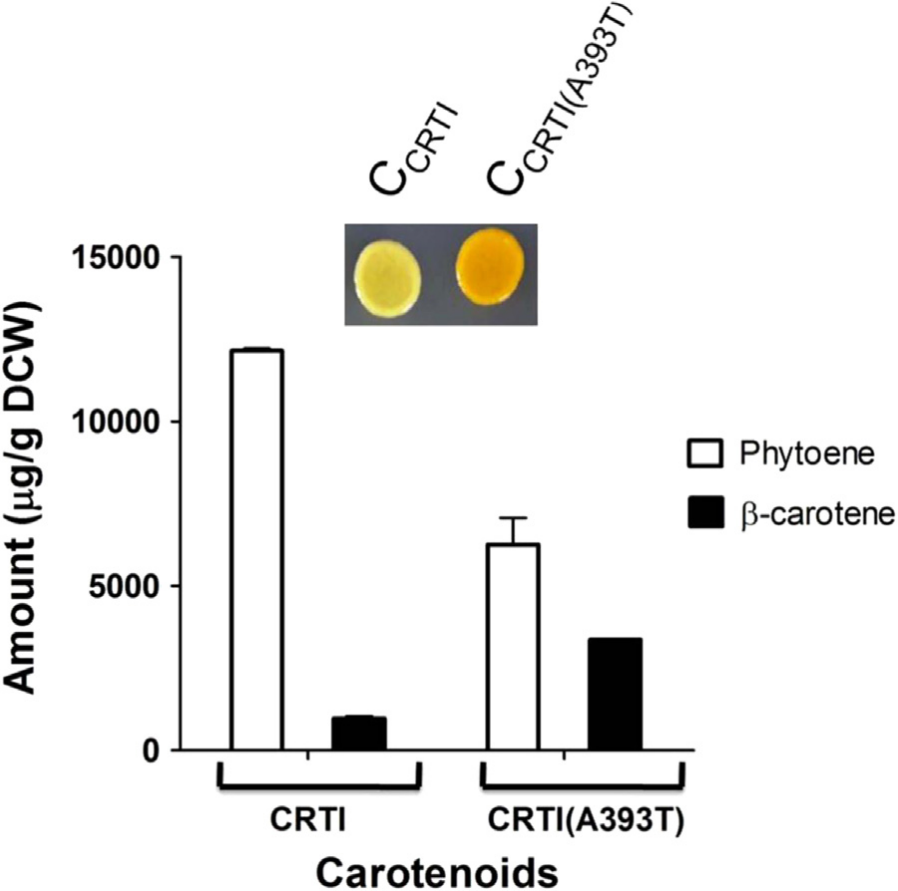
Effect of phytoene dehydrogenase mutant *Rt*CRTI(A393T) isolated by directed evolution on carotenoid levels where *CRTI* and *CRTI(A393T)* expressed under CYC promoter and *GGPPS*, *PSY1* expressed under TEF promoter.Inset: Confluent spot (4* 10^4^ cells) showing pigmentation of both strains.

As we detected a metabolic bottleneck at the rate limiting step of phytoene dehydrogenase, a critical requirement for a successful genetic screen was to overcome this metabolic bottleneck. Two different ways this accumulation of phytoene could be alleviated were (a) by increasing the activity of the rate limiting enzyme phytoene dehydrogenase and (b) by decreasing the precursor levels thereby leading to decreased phytoene levels. Regarding the first possibility where phytoene dehydrogenase activity needed to be increased, one possible approach that has been tried earlier (Verwaal et al., 2007) is to increase the expression levels of the rate limiting enzyme, phytoene dehydrogenease, by either increasing the copy number of the plasmid, or the promoter strength driving expression. However, both these approaches tend to place a higher load on the cells resources. We opted to apply a directed evolution strategy to isolate more active mutants of the rate limiting phytoene dehyrogenase (*Rt*CRTI) by exploiting the pigmentation phenotype. We created a mutagenic library through *in vitro* random mutagenesis of phytoene dehydrogenase in the plasmid pRS314CYC- *Rt*CRTI (where *Rt*CRTI was under the weak CYC promoter).This was a low colour producer that was essential for such a colour based screen, since higher colour leads to a saturation in such visual screens (Wang et al., 2000). The library of *Rt*CRTI mutants in this plasmid was directly transformed into the *S. cerevisiae* strain with TEF_GGPPS_+TEF_PSY1_ plasmids. Transformants were selected on minimal plates and screened on the basis of increased colour as compared to the colour of the starting strain. Six mutants were initially obtained, and after isolation of the plasmids, amplification through *E. coli,* recloning into a fresh vector, and retransformation, three mutants could be confirmed to confer increase pigmentation to the strains. The genes were sequenced and two mutants were found to have an Ala393Thr mutation in the coding sequence, while one mutant was found to have an Ala394Gly mutation. Interestingly, both these mutants clustered in the same region. Modelling the phytoene dehydrogenase of *R. toruloides* on the crystal structure of *Pantoea ananatis* (PDB Id-4DGK) (Schaub et al., 2012) indicated that the residues Ala393 and Ala394 were not present in the active site, and were interestingly, also not conserved in bacterial or fungal enzymes (Data not shown). The subsequent experiments we have worked with *Rt*CRTI_A393T_. To confirm whether the mutation indeed led to increased activity of *Rt*CRTI, we quantitated carotenoids by HPLC and observed that there is 2 fold decrease in levels of phytoene and 3.4 fold increase in β-carotene levels with the mutant enzyme as compared to the WT enzyme (Fig. 3).

### 3.4 Decreasing metabolic precursors to phytoene yields a carotenoid-based phenotypic screen that responds to increased flux in the isoprenoid pathway

**Fig. 4:**
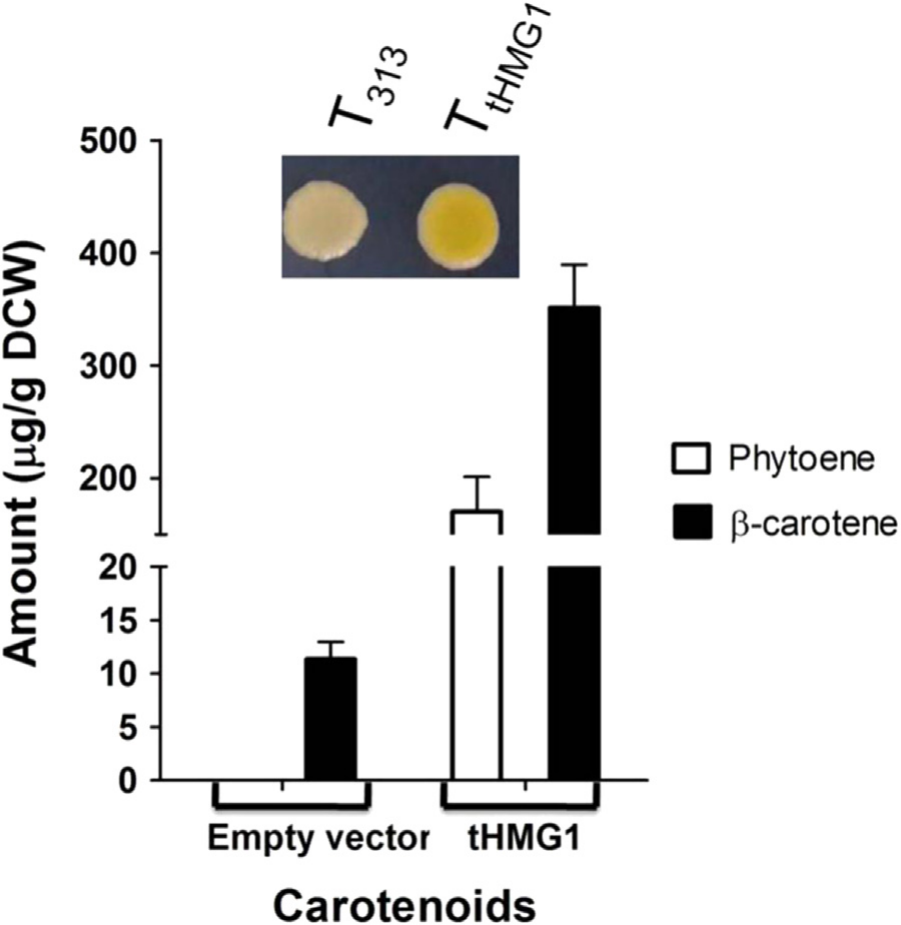
Effect of *tHMG1* over expression on the carotenoid levels in the strain containing *Rt*CRTI_(A393T)_ under GPD promoter and native *BTS1* of *S. cerevisiae* on the carotenoid levels. Inset: Confluent spot (4* 10^4^ cells) showing pigmentation of above strain.

Since the *Rt*CRTI enzyme was revealed to be rate limiting we examined if the more efficient *Rt*CRTI_A393T_ variant was adequate to allow increase in the metabolic flux of isoprenoid (upon over-expression of *tHMG1*). However, surprisingly, it did not show the expected increase in pigmentation with *tHMG1* even with a strong promoter (TEF or GPD) (data not shown). It suggested that phytoene was still accumulating despite use of strong promoter and an active mutant of phytoene dehydrogenase. Chemical analysis of the TEF_GGPPS_+TEF_PSY1_+TEF_CRTI(A393T)_ combination showed that with over expression of *tHMG1*, there is 2 fold increase in production of phytoene, but β-carotene levels decreases by 1.5 fold (Table S3).We therefore considered it necessary to limit the levels of phytoene precursors and thereby prevent phytoene accumulation. This was investigated by employing a less efficient version of upstream gene, GGPP synthase. However, as even the combination of *Rt*GGPPS under the weaker CYC promoter gave only marginal increase in colour, we considered it possible that the *Rt*GGPPS was too efficient and must be replaced by a less efficient enzyme (Jiang et al., 1995). Therefore we attempted to decrease the phytoene levels by using *S. cerevisiae* GGPPS (*BTS1*) under its native promoter in place of *Rt*GGPPS. Using this combination of *S. cerevisiae BTS1* along with *Rt*CRTI_A393T_ under the stronger GPD promoter (pRS315GPD-*Rt*CRTI_A393T_), pRS314TEF-*Rt*PSY1, we could finally observe the desired increase in colour with over expression of *tHMG1* (Fig. 4). This strain also showed a mild growth defect that was also complemented by *tHMG1* over expression suggesting that the GGPP pool becomes limiting in the assay strain. Chemical analysis of this strain combination by HPLC revealed 31-fold increase in β-carotene and also an increase in phytoene levels (Fig. 4). As the levels of β-carotene are significantly higher as compared to phytoene, it is probably reflected in an increased pigmentation. Therefore, this strain combination seems suitable as a visual genetic screen for isolating new genes and mutants that increase the flux in the isoprenoid pathway.

To determine whether the combination of plasmids used in genetic screen described above behaved similarly in other *S. cerevisiae* strain backgrounds, we examined the industrially important strain of *S. cerevisiae*- CEN.PK-1C and transformed it with the above combination of plasmids along with either pRS313TEF or pRS313TEF-*tHMG1.* Our results indicated a similar increase in colour with over expression of *tHMG1* suggesting that the screen combination can be generalised for other *S. cerevisiae* backgrounds (Fig. S2)

### 3.5 Isolation of mutants in *SPT15*, the global TATA binding protein using the phenotypic pigmentation screen that can significantly increase the flux in the isoprenoid pathway

**Fig. 5:**
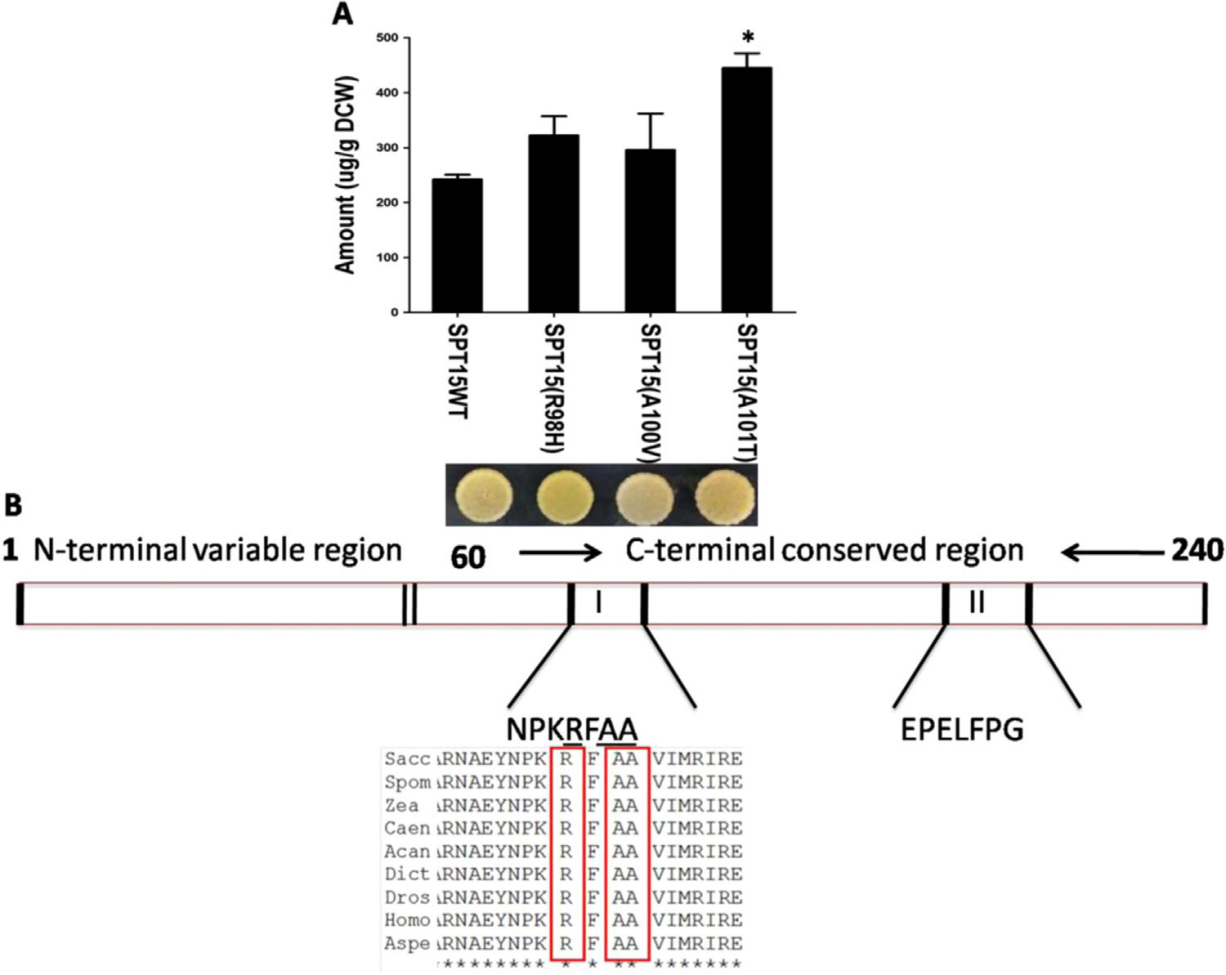
Effect of *SPT15* mutants isolated from genetic screen (A) on the pigmentation spot containing 4* 10^4^ cells and β-carotene levels (B) schematic representation of location of *SPT15* mutant residues. For statistical analysis, *t*-test was performed and spt15 mutants were individually compared to SPT15WT and * represents p value<0.05.

To evaluate the carotenoid-based screen for its ability to identify new genes/mutations that increase flux through the isoprenoid pathway, we decided to examine if mutants in *SPT15* could be isolated that could result in increased flux in isoprenoid pathway. SPT15 functions as a global TATA binding protein (TBP) and thus has an involvement in multiple pathways and networks (Alper et al., 2006) but with no known links to the isoprenoid pathway. *SPT15* was cloned downstream of the TEF promoter and subjected to random *in vitro* mutagenesis with hydroxylamine. The *SPT15* mutant library was transformed into the screen described above. A total of 6 colonies were initially selected on the basis of enhanced colour as compared to control background strain. Plasmids were isolated from these strains, purified, subcloned in fresh vector backbone and then amplified through *E. coli* and retransformed into the yeast strain and serially diluted to confirm the pigmentation phenotype. Three of these mutants from independent mutant stocks were found to display increased pigmentation (Fig. 5A). The other three colonies failed to show increase in colour after subcloning to fresh vector background suggesting that these colonies may be carrying mutations in regions other than the coding region (vector backbone, promoter sequence). Sequencing revealed that these mutants carried mutations Arg98His, Ala100Val and Ala101Thr respectively. The mutant Ala101Thr showed significant enhancement in colour as compared to the control background strain, and the increased β-carotene levels in these mutants was also confirmed by chemical analysis in this strain (Fig. 5A) as well as a strain expressing all 3 genes under the TEF promoter (Fig. S4). Sequence analysis revealed that these residues are present in the C-terminal stirrup region of *SPT15* and conserved across species (Chasman et al., 1993) (Fig. 5B).

We also examined whether combining the *tHMG1* along with the *spt15* mutants could lead to further enhancement in carotenoid levels. We combined the *tHMG1* with *spt15*_*(A101T)*_ in the developed screen but could not find any further increase with this combination (Fig. S3).

### 3.6 The isolated *spt15* mutants, like *tHMG1*, lead to increased levels of the sesquiterpene, α-Farnesene

To investigate whether the isolated *spt15* mutants were increasing the yield of only carotenoids or if they were increasing the overall flux in the isoprenoid pathway in *S. cerevisiae,* we chose to examine an alternative isoprenoid, the sesquiterpene α-Farnesene (which is produced from FPP in the isoprenoid pathway) in *S. cerevisiae*. We expressed the α-Farnesene synthase gene of *A. thialiana* in *S. cerevisiae* downstream of the TEF promoter and quantified the production of α-Farnesene as described in materials and methods.

**Fig. 6:**
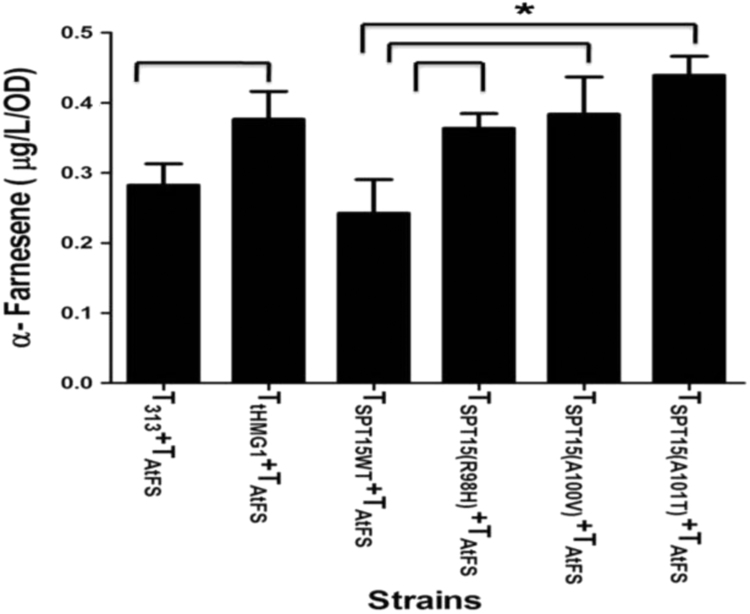
Yield of α- Farnesene obtained in different strain background. For statistical analysis, *t*-test was performed and * represents p value<0.05. T- TEF promoter, *At*FS- α-Farnesene synthase from *Arabidopsis thialana*.

Expression of the α-Farnesene synthase gene of *A. thaliana* produced very little amounts of α-Farnesene (0.29 μg/L/OD_600_) but it was adequate to test the effects of the *spt15* mutants. With over expression of either *tHMG1* or any of the different *spt15* mutants, the yield of α-Farnesene increased upto 1.5 fold. *tHMG1* also led to an approximately similar fold increase in α-Farnesene. The maximum increase in α-Farnesene was observed with over expression of *spt15*_(A101T)_ (0.44 μg/L/OD_600_) (Fig. 6). Importantly, the increase in the levels of α-Farnesene with *spt15* mutants suggests that they are increasing the flux in the isoprenoid pathway and their effects are not exclusive to the carotenoid pathway.

## 4 Discussion

Despite the enormous success in the metabolic engineering of isoprenoids in yeast, a screen for identifying genes/mutations increasing the isoprenoid flux has, surprisingly, been lacking. In this manuscript we have addressed this lacuna, and describe the successful development of a carotenoid-based screen to isolate new genes or mutations that may have an influence in increasing the metabolic flux through the isoprenoid pathway. Although we have carried out visual inspection for identification of mutants in this study, it is also possible to employ more quantitative colorimetric methods as well (Verwaal et al., 2007; Yuan and Ching, 2014). Employing this screen with a candidate gene, *SPT15* that encodes a component of the global transcription machinery, new mutants in *SPT15* have been isolated that can lead to an increased flux in the mevalonate-isoprenoid pathway, thereby revealing a previously unknown metabolic interconnection. This unexpected link is an example of the kind of new insights that such a screen can yield.

In the development of the screen the core carotenogenic biosynthetic genes of the red yeast *Rhodosporidium toruloides* have been used as a source of enzymes which were also codon optimised for *S. cerevisiae* expression. With higher yields of β-carotene and lower levels of phytoene and lycopene relative to earlier reports (Verwaal et al., 2007), these enzymes from *R. toruloides* should be preferred in future studies dealing with carotenoids as screens or products in yeasts. However, despite the expectation that the higher capacity of these enzymes will allow them to pull the isoprenoid flux into the carotenoids without limitations or metabolic bottlenecks, a rate-limiting step at phytoene dehydrogenase was encountered. It is unclear at this stage if the bottleneck at this step that is being repeatedly encountered in *S. cerevisiae*, is unique to *S. cerevisiae* or is encountered in the parent host organisms from which they were obtained.

The study also described more efficient mutants of phytoene dehydrogenase isolated through directed evolution. The exact mechanism by which the *Rt*CRTI mutants could lead to greater product conversion was not investigated. However, it is interesting to note that previous efforts to isolate such mutants with *X. dendrorhous* enzyme have not met with success (Xie et al., 2015).

Evaluation of strains at each developmental step was carried out using *tHMG1*, a known flux increaser. The metabolic bottlenecks could thus be identified and strategies adapted for their alleviation. The final combination which also had reduced phytoene levels also showed a slight growth defect. The growth defect was also overcome by over expression of *tHMG1*, and suggests that the low GGPP pool was responsible for the growth defect in the screen. As GGPP pools of *S. cerevisiae* are utilised for geranyl-geranylation of proteins (Jiang et al., 1995), lower pools might be resulting in slow growth.

The screen enabled us to isolate mutants of the global TATA binding protein SPT15, that could increase the flux in the isoprenoid pathway. SPT15 is part of the cells global transcription machinery and has been used as target for mutagenesis by other groups for improved ethanol tolerance where a triple mutant F177S,Y195H and K218R was identified (Alper et al., 2006) and a spt15-3 with multiple mutations- S136R, K138I, R141G, G147R and K167N and truncation at the N-terminal able to confer oxidative stress tolerance (Zhao et al., 2014) in *S. cerevisiae* were obtained. Interestingly, we were able to get three different mutants of *SPT15*- R98H, A100V, A101T that were not isolated in earlier screens and carried only a single mutation each in the SPT15 gene. The differences in mutants obtained is likely to be a consequence of the difference in selection strategies employed. Based on the crystal structure of SPT15 these mutated residues are part of the stirrup region (95-101aa) between S2 and S3 β-sheets (Bleichenbacher et al., 2003). The stirrup region I of SPT15 docks with the TFIIA β-barrel. This region is important for interactions with TFIIA and in the pre-initiation complex (PIC) assembly at the promoter, and transcription by RNA polymerase II. Thus the mutated residues in this region of SPT15 may alter the docking of TBP with TFIIA, alter its association with other factors, and thereby affect the transcription of several genes. However, this hypothesis, and the downstream genes or pathways that might be affected by these mutated variants of SPT15 awaits investigation.

The ability of the isolated *spt15* mutants to also produce the increased levels of the sesquiterpene α- Farnesene, that branches off from a different point (FPP) in the isoprenoid pathway underlines the potential for identification of true mutants that increase the flux in the isoprenoid pathway. Although the increase in concentration for farnesene were low (1.5 fold) they were comparable for both *tHMG1* and the *spt15* mutants. The low concentration could be because the *A. thaliana* gene has not been optimised for the process.

The successful identification of these new variants of *spt15* that increased flux in the isoprenoid pathway, though important in itself, is an example of the potential that such a screen holds.

## 5 Conclusion

In summary, we have described the successful development of a screen for the detection of increased flux in the isoprenoid pathway in yeast. It opens up several new possibilities that include not only targeting specific genes as has been done here, but investigating deletion libraries, cDNA libraries of other organisms with high isoprenoid flux, and many other variant or mutant libraries. As a consequence new insights are likely to emerge on the links of unknown factors or metabolites to the isoprenoid pathway and its flux. These studies should enable one to obtain a better understanding of the integration of the isoprenoid pathway of yeast into the larger metabolic and regulatory networks.

## Funding

M.W is a Senior Research Fellow from the University Grants Commission, Government of India. AKB is the recipient of a JC Bose National Fellowship (JCB-12–0036) from Department of Biotechnology, Government of India.

## Conflict of Interest

The authors declare that they have no conflict of interest.

## Footnotes

Appendix A Supplementary data associated with this article can be found in the online version at doi:10.1016/j.meteno.2016.05.004.
