# Supplementary material for: A genetic screen for increasing metabolic flux in the isoprenoid pathway of Saccharomyces cerevisiae: Isolation of SPT15 mutants using the screen
Source: Metab Eng Commun. 2016 May 27;3:164–72. doi: 10.1016/j.meteno.2016.05.004 (PMC5779727; doi:10.1016/j.meteno.2016.05.004)
Supplement: Supplementary file 1 — Supplementary material [file mmc1.pdf]

A genetic screen for increasing metabolic flux in the isoprenoid pathway of *Saccharomyces cerevisiae*: Isolation of *SPT15* mutants using the screen

Wadhwa M, Bachhawat AK*

Department of Biological Sciences, Indian Institute of Science Education and Research, Mohali, S.A.S Nagar, Punjab 140306, India.

Corresponding author- Anand Kumar Bachhawat

Email: anand@iisermohali.ac.in

Tel: +91 172 2293154

Fax: +91 172 2240119

Supplementary Tables:

Table S1. List of oligonucleotides and their sequences used in this study

Oligomer	Sequence (5’-3’)
tHMG1 F	GATCGCGGATCCATGGACCAATTGGTGAAAACTGAAG
tHMG1 R	CATGCGCCCGGGTTAGGATTTAATGCAGGTGACG
Spt15 F	GATCGCGGATCCATGGCCGATGAGGAACGTTTAAAG
Spt15 R	CATGCGCTCGAGTCACATTTTTCTAAATTCAC
*At*FS F	GACGTTCTAGAATGCCTAAACGACAGGCTCAAC
*At*FS R	GGCTCGGATCCTTAATTGAGTGGAAGAGGGTGG

Table S2. List of Plasmids used in the study

Plamids	Relevant features
p416TEF-*Rt*PSY1	p416 TEFp-*Rt*PSY1-CYC1t
pRS314TEF-*Rt*CRTI	pRS314 TEFp-*Rt*CRTI-CYC1t
pRS315TEF-*Rt*GGPPS	pRS315 TEFp-*Rt*GGPPS-CYC1t
pRS314TEF-*Rt*CRTI$_{(A393T)}$	pRS314TEFp-*Rt*CRTI$_{(A393T)}$-CYC1t
pRS313TEF-*tHMG1*	pRS313 TEFp-*tHMG1*-CYC1t
pRS313TEF-*SPT15*	pRS313 TEFp-*SPT15*-CYC1t
p416CYC-*Rt*PSY1	p416 CYC1p-*Rt*PSY1-CYC1t
pRS314CYC-*Rt*CRTI	pRS314 CYC1p-*Rt*CRTI-CYC1t
pRS315CYC-*Rt*GGPPS	pRS315 CYC1p-*Rt*GGPPS-CYC1t
pRS314CYC-*Rt*CRTI$_{(A393T)}$	pRS314CYCp-*Rt*CRTI$_{(A393T)}$-CYC1t
pRS314GPD-*Rt*CRTI$_{(A393T)}$	pRS314GPDp-*Rt*CRTI$_{(A393T)}$-CYC1t
pRS313TEF-*SPT15*$_{(R98H)}$	pRS313 TEFp-*SPT15*$_{(R98H)}$-CYC1t
pRS313TEF-*SPT15*$_{(A100V)}$	pRS313 TEFp-*SPT15*$_{(A100V)}$-CYC1t
pRS313TEF-*SPT15*$_{(A101T)}$	pRS313 TEFp-*SPT15*$_{(A101T)}$-CYC1t
pRS315TEF--*At*FS	pRS315 TEFp-*At*FS-CYC1t

All plasmids indicated in table S2 are from this study.

Table S3. Yield of carotenoids in different background strains

Strain Background	β-carotene (µg/g DCW)	Lycopene (µg/g DCW)	Phytoene (µg/g DCW)
T_{GGPPS}+T_{PSY1}+T_{CRTI}+T_{313}	5444±250	34±7	2770±706
T_{GGPPS}+T_{PSY1}+T_{CRTI}+T_{tHMG1}	9673±874	60±0.26	7890±201
T_{GGPPS}+T_{PSY1}+$T_{CRTI(A393T)}$+T_{313}	12938±1007	ND	4823±676
T_{GGPPS}+T_{PSY1}+$T_{CRTI(A393T)}$+T_{tHMG1}	8223±347	ND	9511±310

T-TEF and T_{313}- pRS313TEF, ND- not detected

Supplementary Figures:

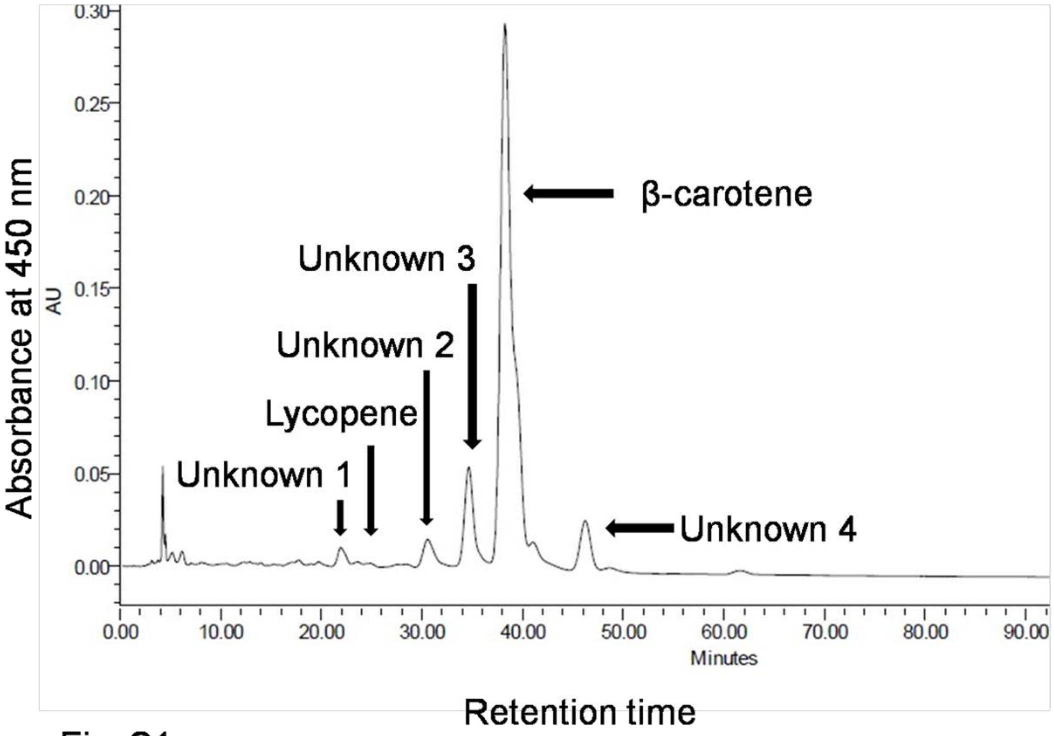

Fig. S1

Fig. S1 HPLC chromatogram of strain ABC 276 containing TEF_{GGPPS} +TEF_{PSY1} +TEF_{CRTI}. The four unidentified peaks are labelled as unknown 1, 2, 3 and 4.

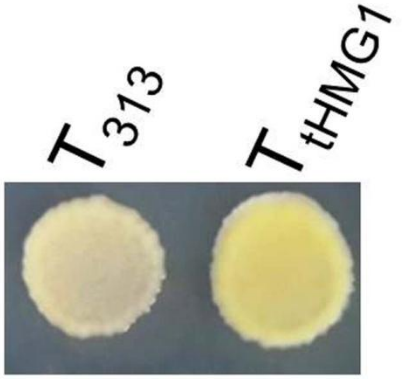

Fig. S2

Fig. S2 Effect of *tHMG1* on pigmentation spot ($4*10^4$ cells) of CEN.PK-1C carrying developed genetic screen combination plasmids p416TEF- *Rt*PSY1, pRS315GPD- *Rt*CRTI$_{(A393T)}$ alongwith either pRS313TEF or pRS313TEF- *tHMG1*.

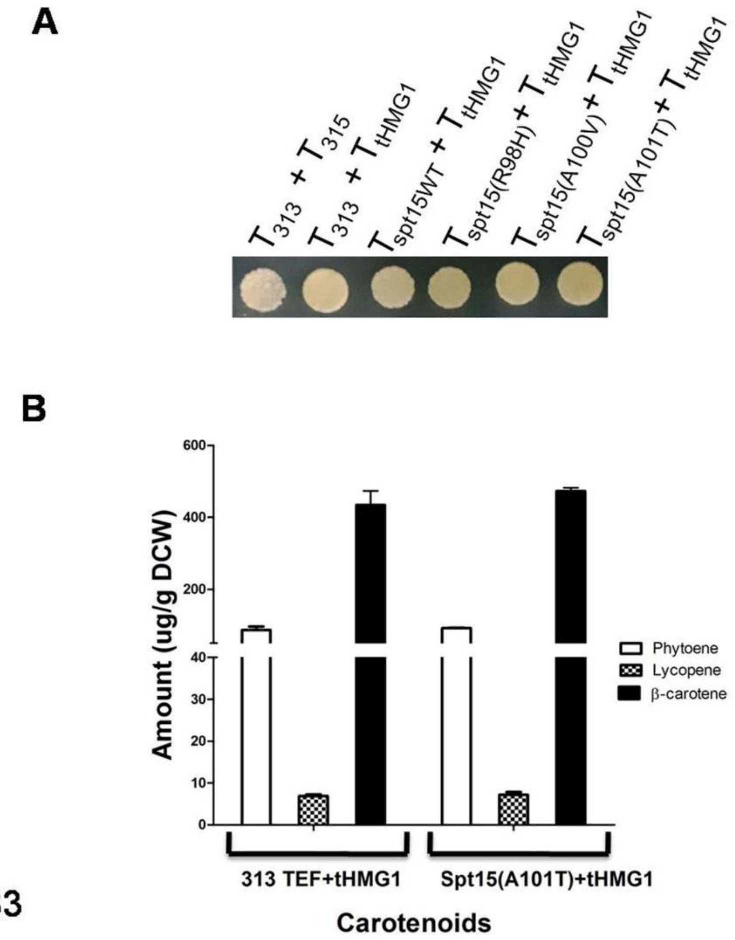

Fig. S3

Fig. S3 Combinatorial effect of over expression of *spt15* mutants and *tHMG1* (A) on the pigmentation spot (4* 10^{4}cells) of genetic assay strain and (b) on the key carotenoids of genetic assay strain

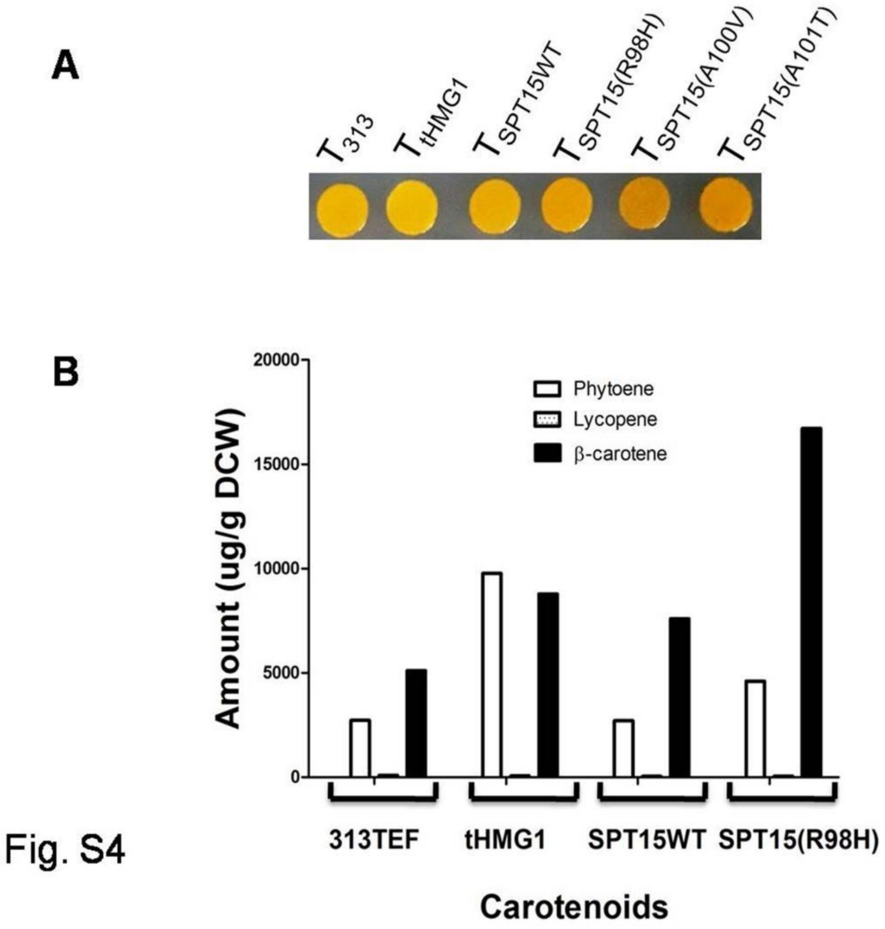

Fig. S4 Effect of *spt15* mutants (A) on pigmentation spots ($4*10^4$ cells) of engineered strain containing T_{GGPPS}+T_{PSY1}+T_{CRTI} (B) on the key carotenoids.
